# Potential causes of malnutrition in older adults in primary healthcare—A cross-sectional study

**DOI:** 10.1016/j.jnha.2025.100745

**Published:** 2025-11-27

**Authors:** Stefan Pfannkuch, Rainer Wirth, Ulrike Trampisch, Dorothee Volkert, Maryam Pourhassan

**Affiliations:** a Department of Geriatric Medicine, Marien Hospital Herne, University-Hospital of Ruhr-University Bochum, Herne, Germany; b Institute for Biomedicine of Aging, Friedrich-Alexander-Universität Erlangen-Nürnberg, Nuremberg, Germany

**Keywords:** Malnutrition, DoMAP model, GLIM criteria, Older adults, Diseases, Symptoms

## Abstract

**Objectives:**

To evaluate the prevalence of the Determinants of Malnutrition in Aged Persons (DoMAP) and identify determinants of malnutrition among older adults attending primary healthcare.

**Design and setting:**

Prospective, observational, monocentric study in primary healthcare.

**Participants:**

500 older adults.

**Measurements:**

Malnutrition was diagnosed using the Global Leadership Initiative on Malnutrition (GLIM) criteria. Potential causes of malnutrition were assessed by the attending physician using the DoMAP model with a 1:1 recruitment of malnourished and non-malnourished older persons.

**Results:**

Malnourished individuals (mean age 81.7 ± 5.0 years; 59% women) exhibited a significantly higher prevalence of almost all DoMAP determinants compared to non-malnourished persons, particularly low intake (88 vs. 11%), high requirements (83 vs. 49%), poor appetite (73 vs. 9%), shopping difficulties (59 vs. 26%), inflammation (81 vs. 49%), gastrointestinal disease (17 vs. 2%), cancer (11 vs. 1%), depression (35 vs. 19%), dementia (21 vs. 6%), polypharmacy (60 vs. 38%), and hospitalization (27 vs. 4%). The mean total determinants count was significantly higher in malnourished participants (14.9 ± 5.0) than in non-malnourished ones (6.8 ± 4.4; *p* < 0.001). Regression analysis revealed low intake as the strongest determinant at Level1; poor appetite, forgetting to eat, shopping difficulties and inflammation at Level2; gastrointestinal disease, cancer and depression at Level3, and frailty and hospitalization at Level4.

**Conclusion:**

This study highlights the complex multifactorial nature of malnutrition in older adults attending primary healthcare, confirming the superior role of low intake and poor appetite among other determinants. The DoMAP model offers a structured framework for potential causative factors of malnutrition in older subjects.

## 1 Introduction

Malnutrition is a prevalent condition among older adults living independently, stemming from a combination of physical, psychological, social and medical changes that can adversely affect nutritional intake and clinical outcomes [1,2]. Maintaining optimal nutritional status and sufficient nutrient intake is crucial for preserving health and quality of life, serving as fundamental aspects of well-being and as key factor in promoting healthy aging. Nonetheless, older individuals are particularly vulnerable to nutritional challenges that may lead to malnutrition due to various underlying mechanisms [3,4].

A major contributor to malnutrition in in this population is reduced food consumption, often linked with increased metabolic demands associated with underlying illnesses [5]. Further, a decline in nutritional intake can be attributed to age related changes, such as diminished hunger and altered satiety control, changes of gastrointestinal motility and reduced sensory capacity, notably taste and smell, leading to what is known as the "anorexia of aging" [6,7]. Contributing factors also include diseases, compromised dental health, negative side effects from medications, cognitive impairment, and socio-emotional challenges such as isolation and depression. Functional impairment such as difficulties with shopping, meal preparation, or mobility limitations can lead to loss of autonomy and further exacerbate the risk of malnutrition among older adults living at home. These multifactorial mechanisms are especially relevant in the context of older patients presenting at a general practitioner’s office, who often represent a heterogeneous and vulnerable group within the community. While they may be classified as community-dwelling, many face complex medical and social challenges that elevate their nutritional risk. Yet, the full dynamics of malnutrition in this setting remain only partially understood. A comprehensive understanding of the underlying causes is essential to develop effective strategies for prevention and intervention.

Studies determining the prevalence of malnutrition in community-dwelling older adults are scarce. A meta-analysis of studies utilizing validated screening instruments estimates the prevalence at approximately 8.5% in this population [8]. Pooled data from five European longitudinal aging cohorts further reveal that up to 10.8% of older adults have a low body mass index (BMI < 22 kg/m²), while 2.3% to 8.0% experience unintentional weight loss exceeding 3 kg within three months in community-dwelling older adults [9]. These indicators reflect a significant burden of nutritional compromise among individuals living independently. Importantly, malnutrition is often already present before clinical contact occurs; many older adults exhibit nutritional deficits prior to hospitalization or institutionalization [10,11]. Nevertheless, in community settings, malnutrition remains underdiagnosed and undertreated, and a comprehensive evaluation of its contributing factors is still lacking.

A systematic review of studies in community‐dwelling older adults highlighted 122 potential protein‐energy malnutrition determinants across 28 studies [12]. To build upon this evidence, we applied the “Determinants of Malnutrition in Aged Persons” (DoMAP) model which was developed within the European Knowledge Hub “Malnutrition in the Elderly (MaNuEL)” through a multistage consensus process using a modified Delphi method, involving 33 international experts in geriatric nutrition [4]. The DoMAP model offers a structured framework that contributes to a common understanding of the multifaceted factors and potential causative mechanisms underlying malnutrition in older persons. Using the DoMAP framework, this study aimed to quantify the prevalence of its specified malnutrition causes and to compare their distribution between malnourished and non‐malnourished community-dwelling older adults, thereby identifying the most influential determinants.

## 2 Subjects and methods

This is a prospective, observational, monocentric outpatient study conducted from December 2022 to March 2024. Community-dwelling older adults were recruited during routine clinical visits at a general practitioner’s office in Mülheim, Germany (*n* = 500). The study aimed to enrol approximately 50% of participants with and without malnutrition. Eligible participants were those aged 75 years or older who could provide a reliable weight measurement. Exclusion criteria included terminal illness, acute fluid imbalance (such as decompensated heart failure or severe dehydration), patients requiring hemodialysis and patients requiring artificial nutrition for more than two weeks, and amputated limbs. Eligible participants were informed verbally and in writing by the first author, a trained medical specialist, about the study’s purpose, procedures, and content, and provided written informed consent. All research-related data, including the determinants of the DoMAP model and the GLIM criteria, were also collected and documented by the first author.

### 2.1 Assessment and definition of malnutrition

Malnutrition was evaluated according to the Global Leadership Initiative on Malnutrition (GLIM) criteria [13,14]. All participants underwent initial nutritional screening using the Mini Nutritional Assessment Short Form (MNA-SF). However, the GLIM criteria were applied independently of the screening results to ensure an objective assessment of nutritional status according to the GLIM. This included three phenotypic criteria: non-voluntary weight loss of more than 5% within the past six months or over 10% beyond six months, a body mass index (BMI) less than 22 kg/m², and reduced muscle mass. In this study, we relied exclusively on BMI and the unintentional weight loss criterion, as the assessment of muscle mass was not sufficiently reliable. The etiologic criteria included reduced food intake or assimilation, defined as less than 50% of energy requirements within the last week or any reduction for more than two weeks, the presence of any chronic gastrointestinal condition that adversely affects food assimilation or absorption, and the presence of an inflammatory condition. Participants were also queried about the amount and the timeframe of weight loss, categorizing the duration into intervals of three months, six months, and more than six months. Due to a known prevalence of malnutrition in community dwelling older adults below 10%, the study stratified to enrol an approximately equal number of malnourished and non-malnourished participants in accordance with the study protocol.

### 2.2 Determinants of malnutrition

Malnutrition determinants were evaluated using the DoMAP model, a structured framework designed by experts to harmonise understanding determinants of malnutrition in this population [4]. The DoMAP model is structured in three interlinked, triangle-shaped levels, with malnutrition positioned at the center. Level 1 focuses on the core causes of malnutrition including low intake, high requirements, and reduced nutrient bioavailability. Level 2 outlines 16 direct contributing factors such as dysphagia and poor appetite, which may lead to low intake; diarrhoea, affecting nutrient bioavailability, and increased metabolic rate, which raises nutritional requirements. Level 3 includes 25 indirect contributing factors that influence nutritional status by triggering one or more direct mechanisms outlined in Level 2; e.g. stroke, which can cause dysphagia thereby affecting intake, and medication that might induce nausea and vomiting, with impact on nutrient bioavailability and intake. Surrounding factors (level 4) encompasses broader contextual and age-related factors that further increase the risk of malnutrition, acting in a more subtle and long-term manner such as polypharmacy, multimorbidity, anorexia of aging, low education, frailty and hospitalization.

For several items in the DoMAP model that were not self-explanatory, specific definitions were provided directly on the assessment sheet used to record prevalent conditions. Multimorbidity was defined as the presence of three or more chronic diseases. Polypharmacy was defined as the regular use of five or more different medications, excluding vitamins and mineral supplements. Anorexia of aging referred to a reduction in appetite not attributable to an identifiable medical condition. Age-related functional decline was defined as a loss of function not explained by a single underlying disease. Hospitalization was defined as any hospital admission within the past 365 days. Stroke included both current stroke and previous stroke with residual symptoms. Cancer was defined as an active malignant disease. Surgery referred to a recent operative procedure occurring within the last three months. Medication was considered relevant if adverse side effects were suspected. Restrictive diet was defined as adherence to a dietary regimen limiting energy, carbohydrate, or fat intake.

### 2.3 Geriatric assessment

Frailty was measured using the Clinical Frailty Scale (CFS), which rates participants' functional and activity level on a scale from 1 (very fit) to 9 (terminally ill), with a score of ≥6 indicating frailty [15]. Nutritional status was additionally evaluated using the MNA-SF [16], which assigns a maximum score of 14. Scores ranging from eight to eleven indicated a risk of malnutrition, while scores below eight were classified as malnutrition. It is worth mentioning that geriatric assessment in general practitioners’ offices is limited to selected patients. However, CFS and MNA-SF were documented in each patient recruited for this study.

### 2.4 Statistical analysis

The statistical analysis was performed using SPSS Statistics for Windows (Version 29.0, IBM Corp, Armonk, NY, USA). Due to the lack of robust data for a definitive sample size calculation, certain assumptions were necessary. Assuming the inclusion of low-prevalence risk factors, such as Parkinson’s disease with an estimated prevalence of 0.02 in the group without malnutrition and 0.06 in the malnourished group at an alpha level of 5% (using a two-sided Fisher’s exact test), the calculated total sample size was n = 425. To ensure sufficient differentiation for such low prevalence risk factors and account for potential dropouts, a minimum enrolment of 500 participants from outpatient settings was targeted.

Continuous variables are presented as means and standard deviations (SD) for normally distributed data and as medians with interquartile ranges (IQR) for non-normally distributed data, while categorical variables are reported as absolute numbers and percentages. Comparisons of malnourished and non-malnourished individuals for categorical variables were conducted using the Pearson Chi-square test.

To quantify the overall burden of determinants, each participant’s determinants as defined by the DoMAP model were summed to create a total determinant count, as well as separate counts for each level. An independent samples *t*-test was then employed to compare both the mean total determinant count and the level-specific counts between malnourished and non-malnourished individuals.

In addition, to further examine the interrelationships among the levels of the DoMAP model, partial correlation analyses were conducted to assess the extent to which the determinants at each level are associated with one another.

Binary logistic regression analyses were performed separately for each DoMAP level, with malnutrition status (yes/no) as the dependent variable and variables from each level of the DoMAP model as independent predictors. Results from the regression analyses are expressed as regression coefficients (B), standard errors (SE), odds ratios (Exp(B)) with 95% confidence intervals (CI), and corresponding P values, with statistical significance set at *P* < 0.05.

## 3 Results

Baseline characteristics of the study population are summarized in Table 1. The cohort consisted of 500 older patients visiting a general practitioners office, mainly consisting of community dwelling older adults. However, a total of 128 participants (25.6%) were dependent on home visits, and 29 individuals (5.8%) were living in nursing homes. The mean age was 81.7 years (SD = 5.0); 59% were female. According to the GLIM criteria, 50% of participants were classified as malnourished, as intended by the recruitment stratification. A total of 63% of participants reported previous unintentional weight loss, with a mean weight loss of 6.0 ± 6.3 kg. Among these individuals, 50% experienced weight loss more than six months prior to assessment, 18% within the past three months, and 7% within the past six months.

**Table 1:** Characteristic of study population on admission.

	All (*n* = 500)	Non-malnourished (*n* = 248)	Malnourished (*n* = 252)	P value*
Gender				
Female, *n* (%)	297 (59)	138 (56)	159 (63)	0.122
Male, *n* (%)	203 (41)	110 (44)	93 (37)	
Age (y), mean ± SD	81.7 ± 5.0	81.5 ± 4.8	81.9 ± 5.2	0.384
Height (m), mean ± SD	1.64 ± 0.09	1.65 ± 0.09	1.63 ± 0.08	0.032
Body weight (kg), mean ± SD	73.3 ± 16.5	79.4 ± 15.5	67.4 ± 15.3	<0.001
BMI (kg/m^2^), mean ± SD	27.0 ± 5.3	28.9 ± 4.9	25.1 ± 5.1	<0.001
Previous weight loss				
No, *n* (%)	168 (37)	158 (76)	10 (4)	<0.001
Yes, *n* (%)	287 (63)	49 (23)	238 (96)	
Previous weight loss (kg), mean ± SD	6.0 ± 6.4	1.2 ± 2.7	10.0 ± 5.8	<0.001
Duration of previous weight loss (m)				
<3 months	28 (6)	7 (3)	21 (9)	<0.001
<6 months	31 (7)	3 (1)	28 (11)	
>6 months	228 (50)	39 (19)	189 (76)	
Number of total determinants, mean ± SD	10.9 ± 6.2	6.8 ± 4.4	14.9 ± 5.0	<0.001
Geriatric assessment				
MNA-SF, median (IQR)	12 (9−14)	14 (12−14)	9 (7−11)	<0.001
Normal nutritional status, *n* (%)	261 (52)	213 (86)	48 (19)	<0.001
Risk of malnutrition, *n* (%)	173 (35)	35 (14)	138 (55)	
Malnourished, *n* (%)	66 (13)	0 (0)	66 (26)	
Clinical Frailty Scale, median (IQR)	5 (4−5)	4 (3−5)	5 (4−6)	<0.001
Frailty, *n* (%)	119 (24)	28 (12)	91 (36)	<0.001
No Frailty, *n* (%)	381 (76)	220 (88)	161 (64)	

MNA-SF, Mini Nutritional Assessment Short Form.

* Difference between malnourished and non-malnourished participants.

Based on the MNA-SF, 35% of participants were at risk of malnutrition, and 13% were identified as malnourished. Additionally, 24% (*n* = 119) of the cohort were classified as frail according to the Clinical Frailty Scale. The mean total number of malnutrition determinants was 10.9 ± 6.2 in the overall study population.

**Fig. 1:**
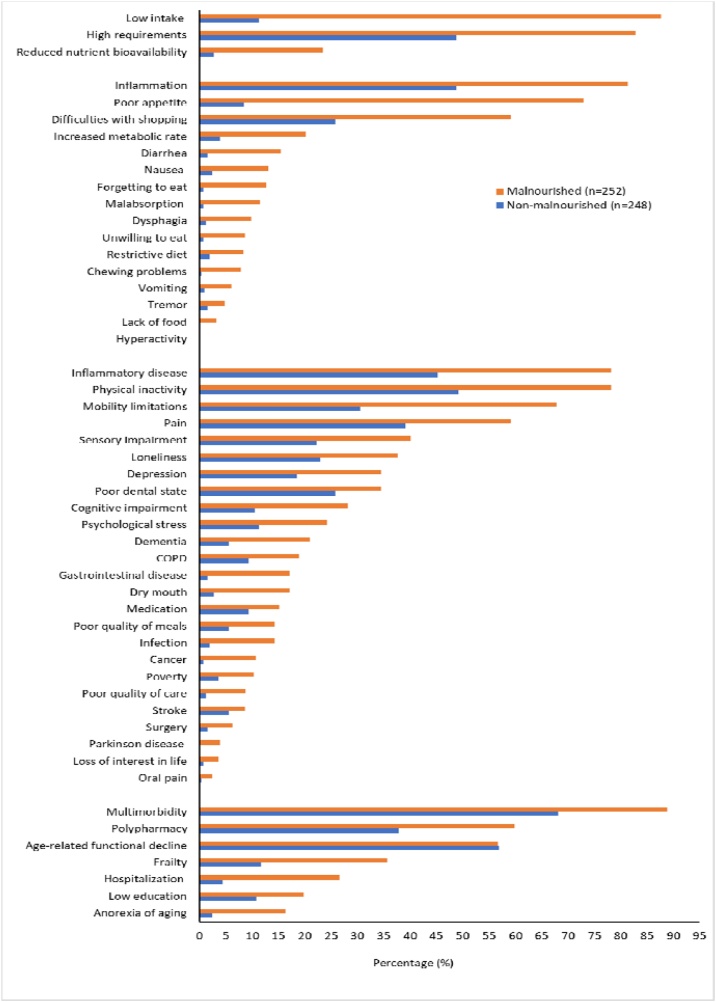
Prevalence and comparison of malnutrition determinants based on the DoMAP model between malnourished (*n* = 252) and non-malnourished (*n* = 248) community-dwelling older adults. The figure illustrates the percentage of participants affected by each determinant, categorized according to DoMAP model levels (Levels 1–4).

Supplementary Tables 1–4 and Fig. 1 provide a detailed overview of the prevalence of malnutrition determinants based on the DoMAP model. The supplementary tables present the frequency of these determinants in the total population and stratified by malnutrition status according to the GLIM criteria, while Fig. 1 illustrates their distribution among malnourished and non‑malnourished individuals. Supplementary Table 1 focuses on level 1 determinants of malnutrition within the DoMAP model. Among malnourished individuals, low intake was the most prevalent determinant (88%), while reduced nutrient bioavailability was the least (23%). In addition, the results show that the proportion of malnourished individuals with reduced nutrient bioavailability, low intake, and high nutritional requirements is significantly higher compared to non-malnourished participants. These findings are visually presented in Fig. 1.

Supplementary Table 2 examine level 2 determinants of malnutrition within the DoMAP model. Among malnourished participants, inflammation was the most prevalent determinant (81%), while hyperactivity was the least common (0%). These results demonstrate that conditions such as malabsorption, diarrhea, nausea, vomiting, dysphagia, chewing problems, lack of food, difficulties with shopping, poor appetite, restrictive diet, unwilling to eat, inflammation, forgetting to eat, hyperactivity and increased metabolic rate were significantly more prevalent in malnourished participants compared to non-malnourished ones (Fig. 1). Tremor was the only determinant that did not differ significantly between both groups.

Supplementary Table 3 explore level 3 determinants of malnutrition within the DoMAP model. Among malnourished individuals, physical inactivity was the most prevalent determinant (79%), whereas oral pain was the least common (2%). While most determinants showed significant differences between malnourished and non-malnourished individuals, medication, stroke, oral pain and loss of interest in life did not exhibit statistically significant differences between both groups (Fig. 1). Supplementary Table 4 addresses Level 4 determinants. Among malnourished individuals, multimorbidity was the most prevalent determinant (89%), while anorexia of aging was the least common (16%). Significant group differences were observed for polypharmacy, multimorbidity, low education, anorexia of aging, frailty, and hospitalization, all of which were more prevalent among malnourished individuals. However, the prevalence of age-related functional decline did not differ significantly between the two groups (Fig. 1).

The total number of determinants was significantly higher among malnourished individuals, with a mean of 14.9 ± 5.0 (out of 51), compared to 6.8 ± 4.4 in the non-malnourished group (*P* < 0.001). This significant difference was consistently observed across all four levels of the DoMAP model. At level 1 (out of 3 factors), malnourished participants had an average of 1.9 ± 0.6 determinants, whereas non-malnourished individuals had 0.6 ± 0.7 (*P* < 0.001). At level 2 (out of 16 factors), the malnourished group had a mean of 3.3 ± 1.6 determinants compared to 1.0 ± 1.1 in the non-malnourished group (*P* < 0.001). At level 3 (out of 25 factors), the average number of determinants was 6.6 ± 2.8 among malnourished individuals and 3.3 ± 2.2 among non-malnourished individuals (*P* < 0.001). At level 4 (out of 7 factors), malnourished participants had a mean of 3.0 ± 1.4 determinants, compared to 1.9 ± 1.3 in the non-malnourished group (*P* < 0.001).

Correlation analyses revealed that determinants from different levels of the DoMAP model were significantly interrelated (for clarity, only results with correlation coefficients greater than *r* > 0.2 are reported here, all *P* < 0.001). For instance, low intake from level 1 was significantly associated with loss of appetite (*r* = 0.421) and unwilling to eat (*r* = 0.233) while reduced nutrient bioavailability was correlated with malabsorption (*r* = 0.777), diarrhoea (*r* = 0.545), vomiting (*r* = 0.321) and nausea (*r* = 0.221). Difficulty with shopping was associated with loneliness (*r* = 0.220), poor quality of meals (*r* = 0.280), and mobility limitation (*r* = 0.248). Forgetting to eat showed correlations with dry mouth (*r* = 0.475), poor dental state (*r* = 0.373), depression (*r* = 0.177), cognitive impairment (*r* = 0.349), and dementia (*r* = 0.446). Unwilling to eat was associated with gastrointestinal disease (*r* = 0.202), medication (*r* = 0.346), poor dental state (*r* = 0.77), dry mouth (*r* = 0.335), and depression (*r* = 0.262). Polypharmacy was associated with medication (*r* = 0.274) and hospitalization (*r* = 0.200).

The results of the binary logistic regression analysis (Table 2), sorted by odds ratios (ORs), demonstrated that several determinants across all four DoMAP levels were independently associated with the presence of malnutrition. At Level 1, all three core mechanisms—low intake, reduced nutrient bioavailability, and increased nutritional requirements—were strongly and independently associated with malnutrition. Among them, low intake emerged as the most influential predictor. At Level 2, poor appetite, restrictive diet, difficulties with shopping, forgetting to eat, diarrhea, dysphagia, inflammation, and increased metabolic rate showed significant associations with malnutrition. At Level 3, malnutrition was significantly associated with gastrointestinal disease, cancer, dry mouth, sensory impairment, mobility limitations, depression, infection, and inflammatory disease. At Level 4, significant associations included anorexia of aging, frailty, multimorbidity, and hospitalization.

**Table 2:** Binary logistic regression analysis of risk factors associated with malnutrition based on the DoMAP model.

Levels of DoMAP model	Malnutrition (yes/no)					
				95% CI for Exp(B)		
	B	Std. Error	Exp(B)	Lower	Upper	P value
Level 1						
Low intake	4.014	0.306	55.346	30.366	100.874	<0.001
Reduced nutrient bioavailability	2.222	0.539	9.225	3.207	26.536	<0.001
High requirements	1.417	0.322	4.127	2.195	7.758	<0.001
Level 2						
Restrictive diet	3.538	0.626	34.413	10.094	117.322	<0.001
Poor appetite	3.200	0.319	24.538	13.128	45.866	<0.001
Forgetting to eat	2.890	0.924	17.986	2.939	110.070	0.002
Diarrhea	2.052	0.784	7.782	1.675	36.153	0.009
Unwilling to eat	1.817	1.070	6.153	0.756	50.077	0.089
Dysphagia	1.730	0.869	5.638	1.027	30.940	0.046
Inflammation	1.325	0.337	3.763	1.944	7.283	<0.001
Malabsorption	1.312	0.972	3.712	0.552	24.949	0.177
Increased metabolic rate	1.123	0.501	3.075	1.153	8.202	0.025
Vomiting	0.763	1.446	2.145	0.126	36.482	0.598
Nausea	0.646	0.708	1.909	0.477	7.643	0.361
Difficulties with shopping	0.585	0.304	1.794	0.989	3.257	0.048
Tremor	0.513	0.992	1.670	0.239	11.672	0.605
Chewing problems	0.225	1.170	1.252	0.126	12.399	0.848
Level 3						
Cancer	2.584	0.821	13.249	2.653	66.173	0.002
Gastrointestinal disease	2.500	0.604	12.186	3.729	39.816	<0.001
Infection	1.811	0.603	6.117	1.877	19.930	0.003
Poor quality of care	1.274	0.734	3.575	0.849	15.059	0.083
Dry mouth	1.215	0.521	3.371	1.213	9.367	0.020
Surgery	1.203	0.730	3.330	0.797	13.918	0.099
Inflammatory disease	0.956	0.264	2.602	1.552	4.364	<0.001
Mobility limitations	0.903	0.290	2.468	1.397	4.357	0.002
Poverty	0.871	0.605	2.390	0.730	7.827	0.150
Cognitive impairment	0.746	0.515	2.108	0.768	5.785	0.148
Depression	0.724	0.329	2.062	1.082	3.933	0.028
Psychological stress	0.678	0.371	1.969	0.951	4.077	0.068
Loss of interest in life	0.673	0.929	1.961	0.318	12.104	0.468
Sensory impairment	0.659	0.275	1.933	1.128	3.312	0.017
Medication	0.650	0.388	1.915	0.894	4.100	0.094
Dementia	0.481	0.619	1.617	0.481	5.438	0.437
COPD	0.373	0.360	1.452	0.717	2.941	0.301
Loneliness	0.369	0.275	1.446	0.843	2.480	0.180
Stroke	0.248	0.467	1.282	0.513	3.200	0.595
Physical inactivity	0.199	0.247	1.220	0.753	1.978	0.420
Oral pain	0.096	1.244	1.100	0.096	12.601	0.939
Poor quality of meal	0.067	0.536	1.069	0.374	3.058	0.900
Pain	−0.246	0.279	0.782	0.453	1.350	0.377
Poor dental state	−0.360	0.287	0.698	0.397	1.226	0.211
Level 4						
Anorexia of aging	2.008	0.477	7.448	2.924	18.973	<0.001
Hospitalization	1.658	0.358	5.251	2.605	10.584	<0.001
Frailty	1.064	0.262	2.899	1.733	4.848	<0.001
Multimorbidity	0.865	0.294	2.374	1.335	4.224	0.003
Polypharmacy	0.267	0.229	1.306	0.834	2.044	0.244
Low education	0.244	0.294	1.277	0.718	2.271	0.405
Age-related functional decline	−0.296	0.213	0.744	0.490	1.129	0.164

As a methodological consideration, lack of food and Parkinson’s disease were excluded from the regression model due to the very small number of affected individuals (*n* = 8 and *n* = 10, respectively). However, including or excluding this variable had no meaningful impact on the overall results, as it remained statistically non-significant for both items. In addition, to evaluate the impact of nursing home residents, we repeated the analyses after excluding these participants. The results remained consistent, with only minor changes in estimated values, and all statistical significance was preserved. This stability is likely attributable to the relatively small proportion of nursing home residents and the predominance of community-dwelling individuals in the overall sample.

## 4 Discussion

Malnutrition in community-dwelling older adults remains a complex and under-addressed geriatric syndrome. Despite slowly growing awareness, comprehensive analyses of its underlying causes are still limited, particularly in this population. Our study demonstrates that most of the determinants of malnutrition in older adults, as outlined by the DoMAP model, significantly differ between malnourished and non-malnourished community-dwelling individuals. Notably, our findings support both the significant associations and the potential mechanistic interrelationships among a broad range of determinants, suggesting that these variables contribute to the development of malnutrition via both direct and indirect mechanisms.

With regard to central mechanisms leading to malnutrition, low nutritional intake emerged as the most prevalent determinant—present in 88.0% of malnourished participants, compared to 83% for high nutritional requirements and 23% for reduced nutrient bioavailability. However, these central mechanisms were as well but less present in non-malnourished subjects, with the biggest difference in low intake. The regression analysis revealed that low nutritional intake was associated with a 55‐fold increased risk of malnutrition, underscoring its role as the most important direct mechanistic contributor in this cohort. This strong association is also partly explained by the fact that low intake constitutes a core component of the GLIM diagnostic criteria. In addition, correlation analyses revealed that low intake was significantly associated with a wide range of other determinants across multiple levels of the DoMAP model, including loss of appetite, unwillingness to eat, increased metabolic rate, infection, inflammatory disease, and hospitalization. These associations illustrate how low intake may result from upstream physiological or contextual conditions, reinforcing its position as both a consequence of other determinants and a direct mechanism leading to malnutrition.

Within level 2 of the DoMAP model, inflammation (81%), poor appetite (73%), and difficulties with shopping (59%) were the most common findings among malnourished participants, all of which contribute to reduced nutritional intake. These factors were also observed in non‐malnourished individuals, though at significantly lower frequencies, reflecting the heterogeneous vulnerability of community-dwelling older adults and suggesting that such symptoms must persist over time to result in weight loss and malnutrition. Notably, poor appetite was associated with a 24‐fold increased risk of malnutrition in the regression analysis, underscoring its role as a major determinant, a strong association that is supported by the literature [12,17]. For example, a systematic review of observational studies in community‐dwelling older adults demonstrated that poor appetite, alongside hospitalization and poor self-reported health represents a critical determinant of malnutrition [12].

Inflammation has been identified as another key determinant in our study, consistent with other research. In a cross-sectional study of 158 healthy community-dwelling adults aged 75–85 years, higher levels of C-reactive protein (CRP ≥ 5.0 mg/L) were significantly associated with moderate appetite, with participants being 3.2 times more likely to report appetite impairment (95% CI: 1.0–10.4, *P* = 0.047) [18]. This suggests that inflammation is a pivotal factor in the development of malnutrition and highlights the importance of considering inflammation as a mediator of disease-related low food intake and malnutrition. Difficulties in shopping emerged as another significant determinant for malnutrition in our study. This finding is supported by previous research; for example, a longitudinal study in Canada, which assessed body weight changes over a 5-year period in 584 community-dwelling and 237 institutionalized older adults, demonstrated that shopping difficulties - arising from a combination of physical, cognitive, and social challenges - were associated with an increased risk of malnutrition [19]. In our analysis, nearly all level 2 determinants were markedly associated with malnutrition, which underscores the diverse and multifactorial etiology of malnutrition and the selection of factors in the DoMAP-model.

In developed countries, disease is frequently cited as a primary cause of malnutrition, given that both acute and chronic disorders can exacerbate nutritional deficits [2]. Analysis of level 3 determinants revealed that gastrointestinal disease, cancer, infection and depression were the most significant determinants associated with malnutrition. Notably, gastrointestinal disease was confirmed as a key contributor, supporting previous evidence that gastrointestinal dysfunction adversely affects nutrient assimilation and overall health in older adults [20,21].

In routine clinical practice, the etiology of weight loss and malnutrition in older adults often differs substantially from that in middle-aged populations, as many malnutrition-related diseases predominantly manifest in advanced age. Although one might anticipate that cancer would play a relatively minor role in an older cohort—given the diverse spectrum of geriatric risk factors—our data contradict this assumption. For instance, a retrospective analysis of 65,000 patients (mean age: 58.6 ± 20.9 years) found that unexpected weight loss was recorded in 63,973 individuals, with 1375 (2.2%) subsequently diagnosed with cancer within two years, suggesting that such weight loss in primary care is associated with a broad range of both early- and late-stage cancer [22]. In our study, 11.0% of malnourished participants had an active cancer disease, and cancer emerged as a significant independent determinant for malnutrition (OR = 13.3, 95% CI: 2.6–66.2, *P* = 0.002), underscoring its importance as a relevant determinant of malnutrition in this population.

In our study, depression emerged as a significant determinant for malnutrition and was closely linked with both unwilling to eat and forgetting to eat. This finding is in line with a prior cross-sectional study of 337 community-dwelling older adults (average age 78.4 years), where a current or past diagnosis of depression was identified as an independent risk factor for malnutrition (OR = 5.1, 95% CI: 2.9–9.20, *P* < 0.001) [23]. In contrast, although dementia and cognitive impairment were more prevalent in malnourished participants but they did not emerge as significant determinants for malnutrition in the regression analyses. Nevertheless, both conditions were strongly correlated with forgetting to eat, suggesting a potential indirect role in the development of malnutrition. Symptoms such as forgetting to eat, recognized as consequence of mild dementia, were also significantly associated with malnutrition in our cohort, emphasizing that malnutrition in older individuals is driven not only by underlying diseases but also by a spectrum of associated symptoms. Importantly, many older adults seen in primary care settings present with mild cognitive impairment or earlystage dementia, which may be overlooked due to the limited routine geriatric assessment conducted in the general practice setting. It is well established that progressive weight loss is a common early manifestation of dementia and typically persists throughout the disease course [24]. Nearly all patients with dementia eventually develop nutritional problems, which are frequently recognized only at advanced stages. Due to the severe consequences for general health, weight loss in dementia should be considered not merely as a symptom, but as a comorbidity that significantly contributes to functional impairment [24].

Hospitalization was identified as another significant determinant for malnutrition in our analysis and was more prevalent among malnourished older adults, a finding that is consistent with prior studies in community‑dwelling populations [12,25]. This association may be explained by a combination of factors, including diseases leading to a hospital stay, reduced appetite, dietary restrictions, and the risk of receiving suboptimal nutritional care during the hospital stay. In our study, hospitalization significantly correlated with loss of appetite, difficulty with shopping, and infection. Notably, it has been reported that a substantial proportion of older adults are already malnourished at admission, and many more develop or exacerbate malnutrition during their hospital stay [26].

It is worth noting that the total determinants count was significantly higher among malnourished participants, approximately eight additional determinants compared to non-malnourished individuals. This result is in line with a previous multisetting study that demonstrated an increased likelihood of malnutrition with a higher number of risk factors across community, hospital, home care, and nursing home settings [27]. Together, these findings highlight the multifactorial etiology and the cumulative impact of multiple risk factors on malnutrition risk in older adults.

This study has several limitations. First, its cross-sectional design does not support establishing causal relationships between the identified determinants and malnutrition. In addition, for the phenotypic criteria of the GLIM framework, we relied exclusively on BMI and unintentional weight loss, as the assessment of muscle mass was not sufficiently reliable. However, this may have led to an underestimation of malnutrition in this context, which is of minor relevance in this study, because we stratified for a 1:1 recruitment of participants with and without malnutrition. Furthermore, the evaluation of geriatric assessment in general practitioners’ offices was limited and focused on key indicators routinely assessed in primary care, omitting other relevant aspects of individual health. Future research employing longitudinal designs is warranted to further elucidate the causal mechanisms underlying malnutrition in community-dwelling older adults. Ultimately, a deeper understanding of these specific factors will enhance early identification of persons at risk and effective interventions related to the causative factors.

## 5 Conclusion

This study highlights the complex multifactorial nature of malnutrition in community‑dwelling older adults, confirming the superior role of low intake, poor appetite, gastrointestinal disease and cancer among other significantly associated determinants in this cohort. Nearly all factors of the DoMAP model demonstrated a significant association with malnutrition, supporting its role as a framework for potential causative factors of malnutrition in older subjects.

## CRediT authorship contribution statement

Conceptualization, S.P., R.W, D.V., and M.P.; data curation and investigation, S.P.; formal analysis, M.P. and S.P.; methodology, M.P., S.P. and R.W, project administration, M.P.; supervision and validation, M.P. and R.W.; writing—original draft, M.P. and S.P.; writing—review and editing, S.P., R.W, U.T., D.V., and M.P. All authors have read and agreed to the published version of the manuscript.

## Ethical standard

The authors declare that the study procedures comply with current ethical standards for research involving human participants in Germany. Ethical approval was obtained from the local ethics committee at the Medical Faculty of Ruhr University (Bochum, Germany) before any data were collected (ethics vote no. 22-7629; date of approval: 14 October 2022). This study was registered in the German Clinical Trials Register with the DRKS-ID: DRKS00030850 (https://www.drks.de/drks_web/setLocale_EN.do).

## Funding

There was no funding source.

## Declaration of competing interest

The authors declare no conflict of interest.
