# Supplementary material for: Potential causes of malnutrition in older adults in primary healthcare—A cross-sectional study
Source: J Nutr Health Aging. 2025 Nov 27;30(1):100745. doi: 10.1016/j.jnha.2025.100745 (PMC12702308; doi:10.1016/j.jnha.2025.100745)
Supplement: Supplementary file 1 [file mmc1.docx]

**Supplementary Table 1.** Level 1 of determinants of malnutrition

| Level 1 | Total population  (n=500) | Non-malnourished  (n=248) | Malnourished  (n=252) | *P value |
| --- | --- | --- | --- | --- |
| Reduced nutrient bioavailability (n, %) |  |  |  |  |
| No | 434 (87) | 241 (97) | 193 (77) | <0.001 |
| Yes | 66 (13) | 7 (3) | 59 (23) |  |
| Low intake (n, %) |  |  |  |  |
| No | 251 (50) | 220 (89) | 31 (12) | <0.001 |
| Yes | 249 (50) | 28 (11) | 221 (88) |  |
| High requirements (n, %) |  |  |  |  |
| No | 170 (34) | 127 (51) | 43 (17) | <0.001 |
| Yes | 330 (66) | 121 (49) | 209 (83) |  |

*Difference between malnourished and non-malnourished participants
