# Supplementary material for: Potential causes of malnutrition in older adults in primary healthcare—A cross-sectional study
Source: J Nutr Health Aging. 2025 Nov 27;30(1):100745. doi: 10.1016/j.jnha.2025.100745 (PMC12702308; doi:10.1016/j.jnha.2025.100745)
Supplement: Supplementary file 2 [file mmc2.docx]

**Supplementary Table 2.** Level 2 of determinants of malnutrition

| Level 2 | Total population  (n=500) | Non-malnourished  (n=248) | Malnourished  (n=252) | P value* |
| --- | --- | --- | --- | --- |
| Malabsorption |  |  |  |  |
| No | 469 (94) | 246 (99) | 223 (88) | <0.001 |
| Yes | 31 (6) | 2 (1) | 29 (12) |  |
| Diarrhea |  |  |  |  |
| No | 457 (91) | 244 (98) | 213 (84) | <0.001 |
| Yes | 43 (9) | 4 (2) | 39 (16) |  |
| Nausea |  |  |  |  |
| No | 461 (92) | 242 (98) | 219 (87) | <0.001 |
| Yes | 39 (8) | 6 (2) | 33 (13) |  |
| Vomiting |  |  |  |  |
| No | 484 (97) | 247 (99) | 237 (94) | <0.001 |
| Yes | 16 (3) | 1 (1) | 15 (6) |  |
| Tremor |  |  |  |  |
| No | 484 (97) | 244 (98) | 240 (95) | 0.072 |
| Yes | 16 (3) | 4 (2) | 12 (5) |  |
| Dysphagia |  |  |  |  |
| No | 472 (94) | 245 (99) | 227 (90) | <0.001 |
| Yes | 28 (6) | 3 (1) | 25 (10) |  |
| Chewing problems |  |  |  |  |
| No | 479 (96) | 247 (99) | 232 (92) | <0.001 |
| Yes | 21 (4) | 1 (1) | 20 (8) |  |
| Lack of food |  |  |  |  |
| No | 492 (98) | 248 (100) | 244 (97) | 0.007 |
| Yes | 8 (2) | 0 (0) | 8 (3) |  |
| Difficulties with shopping |  |  |  |  |
| No | 287 (57) | 184 (74) | 103 (41) | <0.001 |
| Yes | 213 (43) | 64 (26) | 149 (59) |  |
| Poor appetite |  |  |  |  |
| No | 295 (59) | 227 (91) | 68 (27) | <0.001 |
| Yes | 205 (41) | 21 (9) | 184 (73) |  |
| Restrictive diet |  |  |  |  |
| No | 474 (95) | 243 (98) | 231 (92) | 0.002 |
| Yes | 26 (5) | 5 (2) | 21 (8) |  |
| Unwilling to eat |  |  |  |  |
| No | 476 (95) | 246 (99) | 230 (91) | <0.001 |
| Yes | 24 (5) | 2 (1) | 22 (9) |  |
| Forgetting to eat |  |  |  |  |
| No | 466 (93) | 246 (99) | 220 (87) | <0.001 |
| Yes | 34 (7) | 2 (1) | 32 (13) |  |
| Hyperactivity |  |  |  |  |
| No | 500 (100) | 248 (100) | 252 (100) | - |
| Yes | 0 (0) | 0 (0) | 0 (0) |  |
| Inflammation |  |  |  |  |
| No | 174 (35) | 127 (51) | 47 (19) | <0.001 |
| Yes | 326 (65) | 121 (49) | 205 (81) |  |
| Increased metabolic rate |  |  |  |  |
| No | 439 (88) | 238 (96) | 201 (80) | <0.001 |
| Yes | 61 (12) | 10 (4) | 51 (20) |  |

*Difference between malnourished and non-malnourished participants
