# Supplementary material for: Potential causes of malnutrition in older adults in primary healthcare—A cross-sectional study
Source: J Nutr Health Aging. 2025 Nov 27;30(1):100745. doi: 10.1016/j.jnha.2025.100745 (PMC12702308; doi:10.1016/j.jnha.2025.100745)
Supplement: Supplementary file 3 [file mmc3.docx]

**Supplementary Table 3.** Level 3 of determinants of malnutrition

| Level 3 | Total population  (n=500) | Non-malnourished  (n=248) | Malnourished  (n=252) | P value* |
| --- | --- | --- | --- | --- |
| Gastrointestinal disease |  |  |  |  |
| No | 453 (91) | 244 (98) | 209 (83) | <0.001 |
| Yes | 47 (9) | 4 (2) | 43 (17) |  |
| Medication |  |  |  |  |
| No | 439 (88) | 225 (91) | 214 (85) | 0.056 |
| Yes | 61 (12) | 23 (9) | 38 (15) |  |
| Cancer |  |  |  |  |
| No | 471 (94) | 246 (99) | 225 (89) | <0.001 |
| Yes | 29 (6) | 2 (1) | 27 (11) |  |
| Parkinson disease |  |  |  |  |
| No | 490 (98) | 248 (100) | 242 (96) | 0.002 |
| Yes | 10 (2) | 0 (0) | 10 (4) |  |
| Stroke |  |  |  |  |
| No | 464 (93) | 234 (94) | 230 (91) | 0.226 |
| Yes | 36 (7) | 14 (6) | 22 (9) |  |
| Dry mouth |  |  |  |  |
| No | 450 (90) | 241 (97) | 209 (83) | <0.001 |
| Yes | 50 (10) | 7 (3) | 43 (17) |  |
| Poor dental state |  |  |  |  |
| No | 349 (70) | 184 (74) | 165 (65) | 0.041 |
| Yes | 151 (30) | 64 (26) | 87 (35) |  |
| Oral pain |  |  |  |  |
| No | 492 (99) | 247 (99) | 245 (98) | 0.122 |
| Yes | 8 (1) | 1 (1) | 6 (2) |  |
| Pain |  |  |  |  |
| No | 254 (51) | 151 (61) | 103 (41) | <0.001 |
| Yes | 246 (49) | 97 (39) | 149 (59) |  |
| Physical inactivity |  |  |  |  |
| No | 180 (36) | 126 (51) | 54 (21) | <0.001 |
| Yes | 320 (64) | 122 (49) | 198 (79) |  |
| Loneliness |  |  |  |  |
| No | 348 (70) | 191 (77) | 157 (62) | <0.001 |
| Yes | 152 (30) | 57 (23) | 95 (38) |  |
| Poverty |  |  |  |  |
| No | 465 (93) | 239 (96) | 226 (90) | 0.004 |
| Yes | 35 (7) | 9 (4) | 26 (10) |  |
| Poor quality of care |  |  |  |  |
| No | 474 (95) | 245 (99) | 229 (91) | <0.001 |
| Yes | 26 (5) | 3 (1) | 22 (8) |  |
| Poor quality of meals |  |  |  |  |
| No | 450 (90) | 234 (94) | 216 (86) | 0.002 |
| Yes | 50 (10) | 14 (6) | 36 (14) |  |
| Sensory impairment |  |  |  |  |
| No | 344 (69) | 193 (78) | 151 (60) | <0.001 |
| Yes | 156 (31) | 55 (22) | 101 (40) |  |
| Mobility limitations |  |  |  |  |
| No | 253 (51) | 172 (69) | 81 (32) | <0.001 |
| Yes | 247 (49) | 76 (31) | 171 (68) |  |
| Loss of interest in life |  |  |  |  |
| No | 489 (98) | 246 (99) | 243 (96) | 0.063 |
| Yes | 11 (2) | 2 (1) | 9 (4) |  |
| Depression |  |  |  |  |
| No | 367 (73) | 202 (81) | 165 (65) | <0.001 |
| Yes | 133 (27) | 46 (19) | 87 (35) |  |
| Cognitive impairment |  |  |  |  |
| No | 403 (81) | 222 (89) | 181 (72) | <0.001 |
| Yes | 97 (19) | 26 (11) | 71 (28) |  |
| Dementia |  |  |  |  |
| No | 433 (87) | 234 (94) | 199 (79) | <0.001 |
| Yes | 67 (13) | 14 (6) | 53 (21) |  |
| Surgery |  |  |  |  |
| No | 480 (96) | 244 (98) | 236 (94) | 0.010 |
| Yes | 20 (4) | 4 (2) | 16 (6) |  |
| COPD |  |  |  |  |
| No | 429 (86) | 225 (91) | 204 (81) | 0.002 |
| Yes | 71 (14) | 23 (9) | 48 (19) |  |
| Infection |  |  |  |  |
| No | 459 (92) | 243 (98) | 216 (86) | <0.001 |
| Yes | 41 (8) | 5 (2) | 36 (14) |  |
| Inflammatory disease |  |  |  |  |
| No | 190 (38) | 145 (54) | 55 (22) | <0.001 |
| Yes | 310 (62) | 113 (46) | 197 (78) |  |
| Psychological stress |  |  |  |  |
| No | 422 (82) | 220 (89) | 191 (76) | <0.001 |
| Yes | 89 (18) | 28 (11) | 61 (24) |  |

*Difference between malnourished and non-malnourished participants
