# Supplementary material for: Potential causes of malnutrition in older adults in primary healthcare—A cross-sectional study
Source: J Nutr Health Aging. 2025 Nov 27;30(1):100745. doi: 10.1016/j.jnha.2025.100745 (PMC12702308; doi:10.1016/j.jnha.2025.100745)
Supplement: Supplementary file 4 [file mmc4.docx]

**Supplementary Table 4.** Level 4 of determinants of malnutrition

| Level 4 | Total population  (n=500) | Non-malnourished  (n=248) | Malnourished  (n=252) | P value* |
| --- | --- | --- | --- | --- |
| Polypharmacy |  |  |  |  |
| No | 255 (51) | 154 (62) | 101 (40) | <0.001 |
| Yes | 245 (49) | 94 (38) | 151 (60) |  |
| Multimorbidity |  |  |  |  |
| No | 107 (21) | 79 (32) | 28 (11) | <0.001 |
| Yes | 393 (79) | 169 (68) | 224 (89) |  |
| Low education |  |  |  |  |
| No | 423 (85) | 221 (89) | 202 (80) | 0.006 |
| Yes | 77 (15) | 27 (11) | 50 (20) |  |
| Anorexia of aging |  |  |  |  |
| No | 453 (91) | 242 (98) | 211 (84) | <0.001 |
| Yes | 47 (9) | 6 (2) | 41 (16) |  |
| Age-related functional decline |  |  |  |  |
| No | 216 (43) | 107 (43) | 109 (43) | 1.000 |
| Yes | 284 (57) | 141 (57) | 143 (57) |  |
| Frailty |  |  |  |  |
| No | 381 (76) | 219 (88) | 162 (64) | <0.001 |
| Yes | 119 (24) | 29 (12) | 90 (36) |  |
| Hospitalization |  |  |  |  |
| No | 422 (84) | 237 (96) | 185 (73) | <0.001 |
| Yes | 78 (16) | 11 (4) | 67 (27) |  |

*Difference between malnourished and non-malnourished participants
